# 

**Published:** 1888-04

**Authors:** 


### BOOK NOTICES.

THE CENTURY.—Mr. Kennan's Siberian papers, illustrated by Mr. G. A. Frost, who accompanied Mr. Kennan on his trip through Asiatic Russia, will begin in the May *Century*. Their appearance has been deferred on account of the author's desire to group in priliminary papers—the last of which will be in the April *Century*—an account of the conditions and events in Russia directly related to the exile system. This system is now to be minutely described and elaborately pictured; and by way of preface to the first illustrated paper, Mr. Kennan will, in a brief statement, answer the question as to how he came to enter upon his arduous and somewhat perilous investigations, and why he and his companion were accorded such extraordinary facilities by the Russian Government itself. In the April *Century* Mr. Kennan will write of "The Russian Penal Code."

STIMULANTS.—Uses, and how best conserved.

This is a handy little volume by J. M. Emerson, the object of which is to show that the moderate use of our native wines, at meals, is not only harmless, but in many instances highly beneficial to health.

The Author has arrayed many statistics from wine producing countries in support of his position.

